# *Cry1Ac* Transgenic Sugarcane Does Not Affect the Diversity of Microbial Communities and Has No Significant Effect on Enzyme Activities in Rhizosphere Soil within One Crop Season

**DOI:** 10.3389/fpls.2016.00265

**Published:** 2016-03-08

**Authors:** Dinggang Zhou, Liping Xu, Shiwu Gao, Jinlong Guo, Jun Luo, Qian You, Youxiong Que

**Affiliations:** Key Laboratory of Sugarcane Biology and Genetic Breeding, Ministry of Agriculture, Fujian Agriculture and Forestry UniversityFuzhou, China

**Keywords:** *cry1Ac* transgenic sugarcane, risk assessment, gene flow, enzyme activity, functional diversity, community structure

## Abstract

C*ry1Ac* transgenic sugarcane provides a promising way to control stem-borer pests. Biosafety assessment of soil ecosystem for *cry1Ac* transgenic sugarcane is urgently needed because of the important role of soil microorganisms in nutrient transformations and element cycling, however little is known. This study aimed to explore the potential impact of *cry1Ac* transgenic sugarcane on rhizosphere soil enzyme activities and microbial community diversity, and also to investigate whether the gene flow occurs through horizontal gene transfer. We found no horizontal gene flow from *cry1Ac* sugarcane to soil. No significant difference in the population of culturable microorganisms between the non-GM and *cry1Ac* transgenic sugarcane was observed, and there were no significant interactions between the sugarcane lines and the growth stages. A relatively consistent trend at community-level, represented by the functional diversity index, was found between the *cry1Ac* sugarcane and the non-transgenic lines. Most soil samples showed no significant difference in the activities of four soil enzymes: urease, protease, sucrose, and acid phosphate monoester between the non-transgenic and *cry1Ac* sugarcane lines. We conclude, based on one crop season, that the *cry1Ac* sugarcane lines may not affect the microbial community structure and functional diversity of the rhizosphere soil and have few negative effects on soil enzymes.

## Introduction

Controlling plant diseases and insect pests by traditional breeding and modern genetic breeding is the key to achieving food security. Agricultural biotechnologies, particularly transgenic breeding, help us to develop promising methods to enable food security. Sugarcane (*Saccharum* spp. hybrids), with a total of 19.4 million hectares production in the world (Pinto et al., 2005), is the major crop for sugar, as well as a promising industrial raw material for biofuel (Egan et al., 1989). However, one of the major problems of this crop is the susceptibility to insect attack (Ismail, 2013), especially the stem borer (*Diatraea saccharalis* F., Lepidoptera, Cambridae), which affects the plant over the whole growing season and results in substantial yield losses, including reductions to sucrose content and biomass (Weng et al., 2011). Agrochemical control, biological control, and breeding for resistance are the three commonly-used stem borer control strategies (Kfir et al., 2002). As a result of the typical feeding behavior of the larvae that bores into the sugarcane stem, agrochemical control usually spraying insecticides and toxic pesticides on a sugarcane field 5–7 times during a single growing season, which is expensive and potentially harmful to the environment (Arencibia et al., 1997; Chailleux et al., 2013). Biological control using entomophages and entomopathogens like *Trichogramma* spp. and *Beauveria basiana* has been unsuccessful for the control of stem borer in long-term field trials (Arencibia et al., 1997; Chailleux et al., 2013). Breeding for resistance as an approach to stem borer management in sugarcane confers advantages such as inherent control and a low pest density in the field (Kfir et al., 2002). However, breeding for borer-resistance in sugarcane is difficult since the borer resistance trait appears to be absent in the gene pool of sugarcane cultivars (Arencibia et al., 1997). Introduction of the *cry1Ac* gene was shown to be an effective and economic strategy to improve the borer-resistance of sugarcane (Srikanth et al., 2011), similarly to genetically modified (GM) soybean (*Glycine max*) (Valderrama et al., 2007; Karthikeyan et al., 2012), cotton (*Gossypium hirsutum*) (Torres and Ruberson, 2006), corn (*Zea mays*) (Dutton et al., 2003), and other crops (Valderrama et al., 2007; Gatehouse, 2008; Karthikeyan et al., 2012), which contain *Bt* genes (*cry1Ac, cry1Ab, cry1c, cry3Bb1* etc.).

However, whether we should allow GM crops such as sugarcane to become commercialized has been widely debated, primarily for environmental safety considerations. According to the International Service for the Acquisition of Agri-biotech Applications, 175.2 million hectares of GM crops were grown globally in 2013, at an annual growth rate of 3%, with the global hectares of GM crops increasing one hundred and seven-fold since 1996 (James, 2014). Although GM crops are being cultivated with increasing frequency and their area is growing annually, the debate continues. GM crops provide an effective alternative tool for controlling target diseases or insects, but the potential impact on the ecological environment has been an issue of major concern. Therefore, safety assessment is essential and critical (Dale et al., 2002; Dunfield and Germida, 2004; Yu et al., 2011).

The majority of concerns regarding the risks associated with GM crops are related to the potential risk to the environment, including gene flow and non-target effects which indirectly impact the diversity of crops (Dale et al., 2002; Dunfield and Germida, 2004; Yu et al., 2011). Gene flow can occur via pollen and seed dispersal to populations of related crops, weeds, and wild relative species, and may also spread via food chains to pollinators, pest natural enemies, mammalians, and microbes (Messeguer et al., 2001; Lu and Snow, 2005; Chandler and Dunwell, 2008). Though ecologists expect the environmental consequences of gene flow from GM crops to be negligible or neutral, possible consequences of gene flow from GM crops are often cited as a major environmental concern (Messeguer et al., 2001; Lu and Snow, 2005; Chandler and Dunwell, 2008). Sugarcane is propagated by rooting of stalks or micropropagation *in vitro*, and its crossing and blossom are limited to special sites because of the rigorous illumination and temperature requirements; for example, Yacheng, Hainan province or Ruili, Yunnan province are currently the only two suitable sites for sugarcane crossing in China (Chen et al., 2011). In exceptional years, sugarcane blossom may occur at some other sites, but the pollen is sterile. Therefore, horizontal gene transfer with associated microorganisms in rhizosphere soil is the most likely way for gene flow to occur from GM sugarcane (Chen et al., 2011; Hussain et al., 2011).

Soil is an essential element for plant growth, and microorganisms in the rhizosphere play a major role in nutrient transformations and element cycling (Dunfield and Germida, 2004; Hussain et al., 2011; Li X. et al., 2014; Turrini et al., 2015). Andow and Zwahlen (2006) detected cry1Ab protein in the rhizosphere soil, transported via root exudates and also found that GM crops could possibly affect rhizosphere and soil communities. Therefore, it is useful to address questions related to soil biodiversity and soil ecosystem functioning under GM crops, and risk assessment of the soil under GM crops should be considered as an important part of transgenic safety evaluation (Dunfield and Germida, 2004; Griffiths et al., 2007; Hussain et al., 2011; Wu et al., 2014).

Considerable previous work has been done in terms of biosafety on the soil enzyme and/or microbial community structure of *cry1Ac* transgene crops, including *cry1Ac* transgenic cotton (Shen et al., 2006; Zhang et al., 2015), *cry1Ac* transgenic brinjal (Singh et al., 2013), and *cry1Ac* transgenic oilseed rape (Liu et al., 2015). Shen et al. (2006) investigated the potential risk of transgenes on the soil ecosystem of *cry1Ac* cotton (Sukang-103) and its non-*cry1Ac* cotton counterpart (Sumian-12) and found that there was no evidence for any adverse effect of *cry1Ac* cotton on the soil ecosystem. Zhang et al. (2015) found that *cry1Ac* cotton did not show any clear effects on soil microbial communities but the microbial communities were markedly affected by the plant growth stage. Singh et al. (2013) evaluated the rhizospheric bacterial community structure of *cry1Ac* brinjal and their near isogenic non-transformed trait and found that microbial biomass carbon showed a slight reduction in *cry1Ac* brinjal soils and the overall impact of c*ry1Ac* expressing transgenic brinjal was lower than that due to seasonal changes. Liu et al. (2015) assessed the impacts of *cry1Ac* transgenic oilseed rape on soil nematodes and microbial communities and concluded that there was no direct effects on the rhizosphere nematode and microbial communities. Similar studies regarding the potential effects on soil ecosystem have been reported on *cry1Ab* rice (Liu et al., 2008), *cry1Ab* maize/corn (Poerschmann et al., 2005; Barriuso et al., 2012) and *cry3Bb* maize/corn (Devare et al., 2004). Liu et al. (2008) found no measurable adverse effect on the key microbial processes or microbial community composition in rhizophere soil of *cry1Ab* rice. Barriuso et al. (2012) found that the cultivation of *cry1Ab* maize during the 4-year period did not change the maize rhizobacterial communities. Devare et al. (2004) revealed that the release of *cry3Bb* corn poses little threat to the ecology of the soil microbial community.

Compared with the other flowering crops, such as rice or sorghum, GM sugarcane belongs to one of the lowest risk plant species when considering food and environment safety because of its flowering mechanisms, vegetative propagation characteristics and the fact that sugar were derived from high temperature boiling process at 107°C. There have been several biosafety reports on transgenic sugarcane lines (Gilbert et al., 2005; Ruan et al., 2007). Gilbert et al. (2005) evaluated the variability in agronomic characteristics and field disease resistance of transgenic sugarcane transformed for resistance to *Sugarcane mosaic virus* (*SCMV*) strain E. Ruan et al. (2007) investigated the effects on enzyme activities and microbe communities in rhizosphere soil of *sugarcane mosaic virus-coat protein* (*ScMV-CP*) transgenic sugarcane and found that there was no change in the soil bacterial diversity and no apparent effect on soil enzyme activities or the population number of soil microbes in the rhizosphere soil. However, little is known about whether the gene flow will occur from *cry1Ac* sugarcane to soil and whether the GM sugarcane will have any unwanted environmental consequences on soil biodiversity and essential ecosystem functioning. In the present study, we evaluated gene flow through horizontal gene transfer to address these concerns. We investigated the effects on structural and functional diversity of microorganisms, along with enzyme activities in soil samples under *cry1Ac* sugarcane and non-GM lines. We also used denaturing gradient gel electrophoresis (DGGE) analysis to assess the bacterial and fungal communities in these soil samples.

## Materials and methods

### Soil and plant material

According to previous reports, the top layer (0–30 cm) of the sugarcane field, which is the cultivated and plough horizon, was recommended as the representative soil sample for biosafety assessment on *cry1Ac* sugarcane (Taylor et al., 2002; Rowell, 2014). Therefore, soil was collected from the top layer (0–30 cm) of the experimental sugarcane field at the Pilot Test Field in Fujian Agriculture and Forest University, Fujian, China. No transgenic sugarcane material had been planted previously in this plot. The soil was air-dried at room temperature, passed through a 1-mm sieve and then homogenized. The methods were adapted from Bao (2000). The soil contained 20.63 g kg^−1^ of total organic carbon (C) content, 0.63 g kg^−1^ of total nitrogen (N), 0.46 g kg^−1^ of total phosphorus (P), 29.9 g kg^−1^ of total potassium (K), 98.3 mg kg^−1^ of available N, 67.5 mg kg^−1^ of available P, and 201.9 mg kg^−1^ of available K.

The donor non-transgenic sugarcane cultivar, FN95–1702 was used as the control. Six *cry1Ac* transgenic sugarcane lines (termed a1, a2, a3, a4, a5, a6 in our tests) contain the synthetic version of the insecticidal 1840 bp *cry1Ac* gene (GenBank: KF630361.1). These six lines were from the parent variety FN95–1702 and were co-transformed *cry1Ac* and *bar* genes via the plasmid *pUBCG0229* through the particle bombardment method. All the above lines were provided by the Key Lab of Sugarcane Biology and Genetic Breeding, Ministry of Agriculture, China.

### Experimental design and soil sampling

Small plot field experiments under natural conditions were conducted in our Pilot Test Field using a completely randomized block design. Five replicates were taken for each line in both the control line FN95–1702 and *cry1Ac* transgenic sugarcane lines. After cultivating young plants in the greenhouse, each 5.2 × 1.1 m field plot was planted with 35 young plants. Growth was consistent and the field management and the effects of irrigation and fertilization were consistent between field plots. The rhizosphere soil (three replicates each sample) was sampled at three main stages in the sugarcane growth period: tillering, elongation and maturing [93, 163, and 253 days (d) after planting, respectively]. The soil sampling method was modified from Shen et al. (2006) and Wei et al. (2012) as follows: Rhizosphere soil from the five sampling sites per block was mixed as a composite rhizosphere soil sample. The soil samples were then sieved using a 2-mm sieve, homogenized and stored at 4°C until further assay. All assays were conduct within 1 month of sampling.

### Gene flow detection by polymerase chain reaction (PCR)

#### Genomic DNA extraction

Total genomic DNA was extracted from 1.0 g of fresh soil sample using the E.Z.N.A®. Soil DNA Kit (Omega Bio-tek, Inc., USA). The DNA quality was assessed by agarose gel electrophoresis and the DNA purity was determined by calculating the A260/A280 ratio using NanoVue Plus™ (GE, New Jersey, USA). The DNA concentrations were also determined by GE NanoVue Plus™ and the final DNA concentrations were adjusted to 50 ng μ L^−1^.

#### Primer design

The sets of primers (*cry1Ac*-3F: 5′-GCTTGGAGCGTGTC TGGGGT-3′, *cry1Ac*-3R: 5′-TTCTGTGGTGGGATTTCGTC-3′, Tm 57°C), of which the amplification product is 610 bp, was used for the specific detection of *cry1Ac.* In addition, the sets of primers (*bar*-1F: 5′-TTTCGGTGACGGGCAGGAC-3′, *bar*-1R: 5′-GCACGAGGCGCTCGGATAT-3′, Tm 63°C and *npt*-2F: 5′-TCCAGCCAGAAAGTGAGG-3′, *npt*-2R: 5′-GGTCGGAAGAGGCATAAA-3′, Tm 53°C) were used for the specific detection of *bar* (GenBank: EU048869.1), and *npt*II (GenBank: M18327.1), of which the amplification products were 140 and 516 bp, respectively. All the primers, of which the purity was of high performance liquid chromatography grade, were synthesized by TaKaRa Biotechnology Co., Ltd., Dalian, China. All primer pairs were checked by PCR and gel extraction, and their PCR products were sequenced by Sangon Biotech, Shanghai, Co., Ltd.

#### PCR program

The optimized PCR reaction was carried out in a 25 μL volume, including 2.5 μL 10 × Ex-Taq Buffer (Mg^2+^ Plus; TaKaRa Biotechnology Co., Ltd., Dalian, China), 0.005 mM each dNTP (TaKaRa Biotechnology Co., Ltd., Dalian, China), 0.005 μM each primer, 0.625 U Ex-Taq DNA polymerase (TaKaRa Biotechnology Co., Ltd., Dalian, China), and 1.0 μL template DNA. The PCR was performed in a thermal cycler (Mastercycler Gradient 96, Eppendorf, Germany) with the following program: an initial denaturation at 94°C for 4 min, 35 cycles of denaturation at 94°C for 30 s, annealing at 57/63/53°C for *cry1Ac*/*bar*/*npt*II for 30 s, extension at 72°C for 30 s, and a final extension at 72°C for 4 min. Then the PCR products were detected by electrophoresis on 1.5% agarose gels stained with ethidium bromide for 1 h at 100 V. All detection assay were performed three times.

### Microbial community diversity by the classic plate counting method

Bacteria, actinomyces and fungi in fresh soil samples were cultured in beef extract, peptone medium, Gause's medium and Martin's medium, respectively. The population number of the culturable microorganisms (colony-forming units; CFUs) in the rhizosphere soil of the *cry1Ac* sugarcane lines and the non-transgenic line were determined using the serial dilution method of plate counting. Three replicates of the inoculated agar plates were incubated at 37°C for 3 d for bacteria, at 28°C for 5 d for fungi, and at 37°C for 5 d for actinomyces, after which colonies were counted (Li et al., 2011).

### Microbial community functional diversity by biolog ecoplate™

Microbial community functional diversity of microorganisms in rhizosphere fresh soil was determined via Biolog EcoPlate™ (Biolog Inc., Hayward, USA). Each fresh soil sample of 5.0 g was shaken in 45 mL of 0.85% (W/V) NaCl for 20 min at 120 r/min^−1^ and then adjusted to a final dilution of 10^−3^. A 150 μL aliquot was inoculated in each microplate well of the 96 wells Biolog EcoPlate™. Then all plates were incubated in darkness at 28°C after covering in polyethylene bags to reduce desiccation. Each sample was processed in triplicate. The rate of C substrate utilization was indicated by the reduction of tetrazolium, a redox indicator dye, which changes from colorless to purple (Wei et al., 2012). The absorbance at 590 nm was measured at 24 h intervals using a microplate reader (Bio-Tek, USA).

### Enzymatic assay

Urease, protease, sucrase, and acid phosphate monoester enzyme activities in rhizosphere soils were determined according to Guan (1986) and Tabatabai (1994) at the three main growth stages (tillering, elongation, and maturing). All determinations of enzymatic activity were performed in triplicate.

### DGGE analysis

Soil total microbial DNA was extracted from soil samples using the MOBIO Ultraclean Soil DNA Isolation Kit according to the manufacturer's instructions manufacturer. For the analysis of soil bacterial diversity and fugal diversity, a 196 bp fragment of the bacterial *16S rDNA* gene and a 390 bp fragment of the fungal *18S rDNA* gene was amplified using primers F338-GC and R534, and FR1-GC and FF390, respectively. PCR was carried out in a volume of 50.0 μL as above, using the following program: an initial denaturation at 95°C for 8 min, 30 cycles of denaturation at 95°C for 30 s, annealing at 55/50°C for *16S rDNA*/*18S rDNA* for 30/45 s, extension at 72°C for 30 s/2 min, and a final extension at 72°C for 10 min. Then the PCR products (2 μL) were detected by electrophoresis on 1.5% (w/v) agarose gels stained with ethidium bromide for 1 h at 100 V to verify that similar concentrations of PCR products had been amplified from each soil sample. The remaining PCR products (45 μL) were then analyzed by DGGE (Bio-Rad D-Code™ Universal Mutation Detection System, Bio-Rad, Shanghai, Co., Ltd.) using a 40–60% denaturing gradient (100% denaturant contained 7 M urea and 40% formamide) on a 6.5% (w/v) polyacrylamide gel for bacterial samples, or a 45–60% denaturing gradient for fungal samples (Vainio and Hantula, 2000). DGGE gels were prepared in advance and were allowed to polymerize for at least 5 h. Gels were run at 80 V and 60°C for 14 h in 1 × TAE (Tris acetate-EDTA buffer) re-circulating buffer for bacterial *16S rDNA* or at 50 V and 60°C for 18 h in 1 × TAE for fungal gels. DGGE gels were stained by silver staining according to the method of Radojkovic and Kušic (2000).

Band quantitative analysis of DGGE gel used the Quantity One band analysis package (Bio-Rad, Shanghai, Co., Ltd.) and statistical analysis used the method described by Fromin et al. (2002).

Bands of interest were excised from the DGGE gel and eluted into a PCR tubes with the sterile distilled water (20 μL). After extraction at 4°C overnight, 2 μL of the solution was used to re-amplify the excised fragment using the same primer pair and PCR conditions as previously described. PCR products were purified from a 1.5% (w/v) agarose gel using the gel extraction kit (Promega), sub-cloned into the pMD19-T vector (TaKaRa) and transformed into competent *E*. *coli* DH5α cells (Tiangen). Sequencing was carried out by Invitrogen Co., Ltd., Shanghai, China. Then the sequences were identified by blast search alignment on the NCBI (National Center for Biotechnology Information). Uncultured/environmental sample sequences were excluded from both fungal and bacterial search parameters. For identification based on blast search homology, the criteria used were consistent similarity at ≥98% to the same species or genus. Sequences identified were submitted to the GenBank database using the submission tool sequin (http://www.ncbi.nlm.nih.gov/Sequin/index.html). All sequences that were sequenced successfully were submitted to GenBank (Accession numbers: KP693619–KP693681). Multiple alignments were made automatically using Clustal X software with minor manual adjustments (Barriuso et al., 2012). Phylogenetic analysis of the aligned sequences was performed using MEGA 5.02. In the neighbor-joining tree generated, the statistical robustness of the tree and the reliability of the branching patterns were confirmed by 1000 bootstrapping replicates (Saitou and Nei, 1987). The other parameters settings were describe as follows: nucleotide sequence evolution model using “maximum composite likelihood,” substitutions to include using “d: transitions+ transversions,” rates among sites using “uniform rates” and pattern among lineages using “same (homogeneous)” (Barriuso et al., 2012). The evolutionary history was inferred using the Neighbor-Joining method (Saitou and Nei, 1987).

### Statistical analysis

Microbial activity in each microplate was expressed as an average of the replicate well-color development (AWCD) to eliminate variation in well-color development caused by different cell densities: AWCD = [Σ (Ci – R)] /31, Where C_i_ is the mean value of the same three wells except for the control well and R is the value of the control well (Wei et al., 2012).

Principal component analysis (PCA; Wei et al., 2012) based on 120-h AWCD data was performed using the SPSS statistical software (SPSS 11.5 for Windows; SPSS, Inc., Chicago, IL, USA). The Simpson's index, Shannon index, Shannon evenness, Brillouin index, and McIntosh index as a way of quantifying the richness and diversity in soil microbial communities were calculated based on Biolog Eco-Plate™ data according to Hackett and Griffiths (1997). The Simpson's index, Shannon index, Shannon evenness of the species diversity of the bacterial and fugal community were also evaluated based on the gray value of the DGGE band (Fromin et al., 2002). The absorption data of the 31 carbon sources in the Biolog Eco-Plate™ were analyzed by PCA for dimensionality reduction. Significant (*P* < 0.05) differences were analyzed by the Tukey's *t*-test with EXCEL 2010, DPS 8.05, and SPSS 11.5. The interaction effects between the sugarcane lines and the growth stages were analyzed using SPSS 11.5 with general linear model analysis (Zeng et al., 2014).

## Results

### Gene flow detection of *cry1Ac* sugarcane by PCR in rhizosphere soil

**Figure 1:**
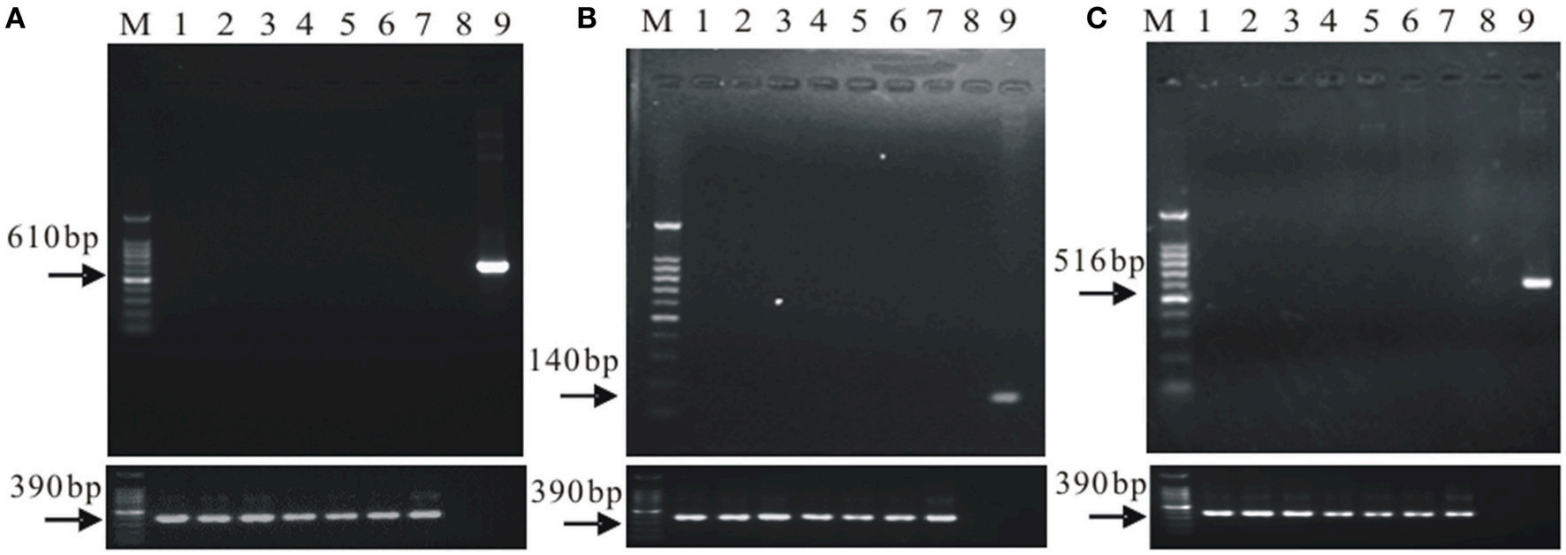
**The gene flow detection of the soil microorganisms in field cultivated the *cry1Ac* transgenic sugarcane. (A)** the *cry1Ac* gene flow detection; **(B)** the *bar* gene flow detection; **(C)** the *npt*II gene low detection. M, 100 bp marker; 1–6, a1, a2, a3, a4, a5, and a6; 7, FN95–1702 (negative control line); 8, ddH_2_O (up panel only); 9, The plasmid 1Ac0229 (positive control, up panel only); upper panel, gene flow detection, lower panel, *18S rDNA*, as control.

The total genomic DNA of the microorganisms in the rhizosphere soil samples was detected by agarose gel electrophoresis (shown in Supplementary Figure 1). Gene flow detection results by PCR are shown in Figure 1.

Results in Supplementary Figure 1 indicate successful extraction of total genomic DNA of the microorganisms in the rhizosphere soil samples. The specific amplification products of *cry1Ac, bar*, and *npt*II were not detected, while all the samples contained the specific band of *18S rDNA* (Figure 1). The results suggest that there is no exogenous gene shifting from the transgenic sugarcane lines to the rhizosphere soil microorganisms.

### Effect of *cry1Ac* sugarcane on microbial community diversity in the rhizosphere soil

The population numbers of the culturable microorganisms in the rhizosphere soil of the *cry1Ac* sugarcane lines are shown in Table 1. The total number of culturable bacteria ranges from 19 to 62 × 10^4^ CFU· g^−1^ dry soil, while the total number of culturable actinomyces and fungi ranges from 26 to 81 × 10^4^ and 0.68 to 1.22 × 10^4^ CFU· g^−1^ dry soil, respectively. The number of culturable microorganisms in rhizosphere soil varied with sugarcane growth stage. The samples of tillering stage and elongation stage presented a higher number of culturable bacteria and actinomyces than those of the maturing stage. This is opposite to finding for the number of culturable fungi. Within each of the growth stages, however, the population numbers of culturable bacteria, or actinomyces and fungi in GM rhizosphere soil showed no significant difference to those in the control line FN95–1702. Based on the analysis of the interaction effects, the growth stage (tillering, elongation, and maturing) significantly affected the population number of the culturable microorganisms including bacteria, actinomyces and fungi (Table 2). Though significant difference of the bacteria diversity was found, no significant difference of the actinomyces and fungi diversity was observed in the interaction effects between the sugarcane lines, regardless of whether the line was *cry1Ac* sugarcane or non-GM (Table 2). Moreover, there were no clear interaction effects between the growth stages and the tested sugarcane lines. The results suggest that the *cry1Ac* sugarcane had no significant effect on the structural diversity of the culturable microbial community in rhizosphere soil.

**Table 1:** **The population number of the culturable microorganisms in the rhizosphere soil of the *cry1Ac* and non-GM sugarcane**.

**Lines for soil samples**		**The population number of the culturable microorganisms (/10**^**4**^ **CFU**· **g**^**−1**^ **dry soil, Mean** ± **SD)**		
		**Bacteria**	**Actinomyces**	**Fungi**
Tillering stage	CK	48.17 ± 7.18ab	57.89 ± 5.92ab	0.22 ± 0.08a
	a1	49.13 ± 5.77ab	57.20 ± 16.22ab	0.26 ± 0.08a
	a2	61.21 ± 19.29a	53.22 ± 0.70ab	0.31 ± 0.08a
	a3	37.71 ± 3.30b	29.38 ± 4.41b	0.24 ± 0.00a
	a4	39.07 ± 1.45b	80.92 ± 18.87a	0.27 ± 0.09a
	a5	62.48 ± 4.24a	72.69 ± 34.27a	0.33 ± 0.07a
	a6	41.65 ± 7.14b	59.96 ± 7.86a	0.22 ± 0.08a
Elongation stage	CK	21.92 ± 3.44a	39.37 ± 9.46ab	0.93 ± 0.31a
	a1	37.79 ± 24.14a	34.54 ± 6.57ab	1.06 ± 0.25a
	a2	24.80 ± 4.28a	42.67 ± 3.08a	1.17 ± 0.49a
	a3	24.96 ± 2.58a	40.70 ± 11.39ab	1.22 ± 0.37a
	a4	36.36 ± 3.43a	31.91 ± 1.40ab	0.93 ± 0.35a
	a5	30.51 ± 3.45a	31.32 ± 3.92b	1.06 ± 0.14a
	a6	22.41 ± 4.29a	31.10 ± 1.85b	1.12 ± 0.09a
Maturing stage	CK	47.13 ± 17.08ab	55.67 ± 23.04a	0.78 ± 0.09ab
	a1	60.67 ± 19.21a	37.12 ± 2.40a	0.75 ± 0.10ab
	a2	59.73 ± 32.14a	48.50 ± 19.84a	0.80 ± 0.05ab
	a3	19.62 ± 4.22b	26.43 ± 8.66a	0.78 ± 0.20ab
	a4	36.09 ± 1.20ab	47.72 ± 12.75a	0.68 ± 0.07b
	a5	44.00 ± 12.60ab	40.36 ± 10.99a	0.85 ± 0.00a
	a6	45.88 ± 1.21ab	47.48 ± 27.54a	0.75 ± 0.03ab

CK, control line FN95–1702; a1, a2, a3, a4, a5, a6: cry1Ac sugarcane lines; values in the column followed by the same letters mean no statistically significant (P < 0.05 level, N = 3).

**Table 2:** **The interaction effects between the sugarcane lines and the growth stages based on the culturable microorganisms in the rhizosphere soil of the *cry1Ac* and non-GM sugarcane using general linear model analysis**.

**Source of variation**	**Bacteria**		**Actinomyces**		**Fungi**	
	***F***	***P***	***F***	***P***	***F***	***P***
Lines	4.071	**0.003**	2.156	0.067	0.659	0.683
Growth stage	16.200	**0.000**	14.054	**0.000**	93.175	**0.000**
Lines × growth stage	1.662	0.111	1.732	0.094	0.339	0.977

Significant P-values (P < 0.05) are indicated in bold type. Lines, FN95–1702 (control line) and cry1Ac sugarcane lines a1, a2, a3, a4, a5, a6. Growth stages: tillering, elongation, and maturing.

### Effect of *cry1Ac* sugarcane on microbial community diversity in rhizosphere soil

**Figure 2:**
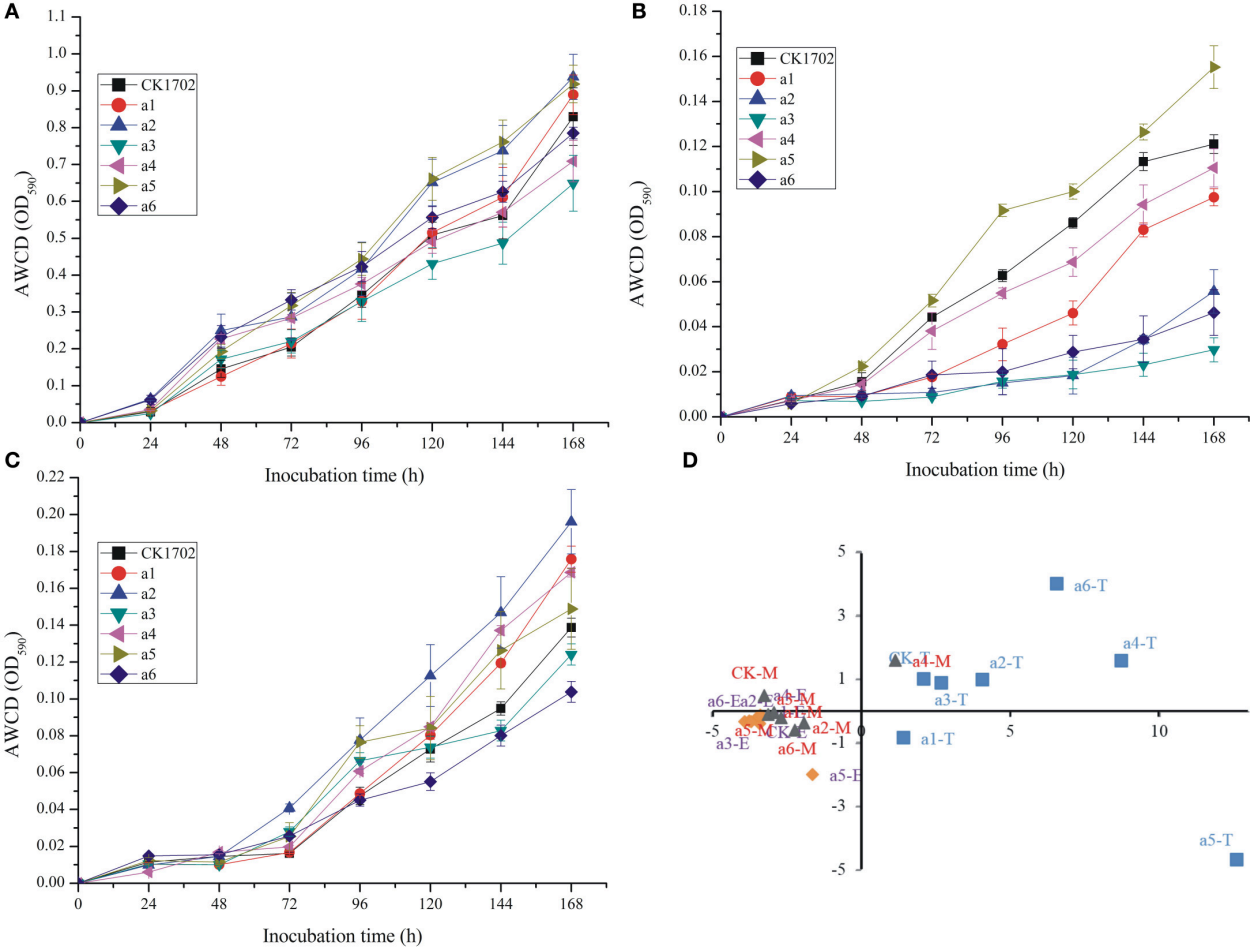
**Effects of *cry1Ac* sugarcane on average well color development (AWCD) during incubation of rhizosphere soil microorganisims and principal component analysis (PCA) based on Biolog EcoPlate results. (A)** AWCD at the tillering stage; **(B)** AWCD at the elongation stage; **(C)** AWCD at the maturing stage. Vertical bars indicate standard error of the means (Mean ± SE, *n* = 3). **(D)** PCA; T, the tillering stage; E, the elongation stage; M, the maturating stage. CK, FN95–1702 (control line). a1, a2, a3, a4, a5, a6: *cry1Ac* sugarcane lines.

Biolog EcoPlate™ is a rapid and effective method to distinguish spatial and temporal changes in microbial metabolic diversity, used previously to evaluate the effect of GM plants on soil. In the present study, no significant difference between GM and non-GM sugarcane were found in AWCD curves at tillering, elongation, and maturing stages (Figure 2).

In addition, no significant difference was found between GM and non-GM sugarcane using the Shannon, Simpson, McIntosh, and Evenness indices (Table 3). These results were also confirmed by PCA (Figure 2D). Although significant differences were observed between different growth stages, the results of the PCA indicated that the carbon source utilization patterns of *cry1Ac* sugarcane and non-transgenic lines were similar at the same growth stage; the control line, and *cry1Ac* sugarcane cluster together at the same stage (expect a5 at tillering stage, and a4 at mature stage; Figure 2D).

**Table 3:** **Effects on the functional diversity index of microorganisms in rhizosphere soil of the *cry1Ac* and non-GM sugarcane**.

**Periods**	**Lines**	**Functional diversity index (Mean ± SD)**					
		**Simpson's index**	**Shannon index**	**Shannon evenness**	**Brillouin index**	**McIntosh index**	**Simpson's index**
Tillering stage	CK	0.785±0.013b	2.357±0.043b	0.912±0.017b	2.349±0.043b	0.546±0.014b	0.785±0.013b
	a1	0.799±0.012b	2.409±0.049b	0.932±0.019b	2.402±0.048b	0.561±0.014b	0.799±0.012b
	a2	0.793±0.006b	2.395±0.034b	0.927±0.013b	2.388±0.034b	0.553±0.006b	0.793±0.006b
	a3	0.824±0.003a	2.542±0.012a	0.983±0.005a	2.533±0.013a	0.590±0.003a	0.824±0.003a
	a4	0.829±0.003a	2.563±0.011a	0.992±0.004a	2.558±0.011a	0.593±0.003a	0.829±0.003a
	a5	0.828±0.005a	2.562±0.023a	0.991±0.009a	2.559±0.023a	0.592±0.006a	0.828±0.005a
	a6	0.799±0.005b	2.398±0.050b	0.928±0.020b	2.393±0.050b	0.559±0.005b	0.799±0.005b
Elongation stage	CK	0.762±0.054ab	2.341±0.167ab	0.906±0.065ab	2.294±0.162ab	0.539±0.062ab	0.762±0.054ab
	a1	0.810±0.031a	2.475±0.134a	0.958±0.052a	2.430±0.121a	0.592±0.041a	0.810±0.031a
	a2	0.834±0.001a	2.575±0.005a	0.996±0.002a	2.512±0.005a	0.625±0.002a	0.834±0.001a
	a3	0.829±0.008a	2.554±0.032a	0.988±0.013a	2.491±0.030a	0.618±0.012a	0.829±0.008a
	a4	0.750±0.073ab	2.295±0.223ab	0.888±0.086ab	2.256±0.213ab	0.525±0.078ab	0.750±0.073ab
	a5	0.676±0.032b	1.992±0.088b	0.771±0.034b	1.979±0.088b	0.441±0.028b	0.676±0.032b
	a6	0.783±0.071ab	2.409±0.219a	0.932±0.085a	2.356±0.205ab	0.565±0.079ab	0.783±0.071ab
Maturing stage	CK	0.784±0.009a	2.378±0.037a	0.920±0.014a	2.345±0.037a	0.557±0.010a	0.784±0.009a
	a1	0.767±0.013a	2.344±0.040a	0.907±0.015a	2.305±0.039a	0.541±0.014a	0.767±0.013a
	a2	0.782±0.026a	2.370±0.101a	0.917±0.039a	2.349±0.099a	0.549±0.028a	0.782±0.026a
	a3	0.769±0.010a	2.319±0.034a	0.897±0.013a	2.293±0.033a	0.537±0.010a	0.769±0.010a
	a4	0.800±0.004a	2.387±0.017a	0.923±0.007a	2.377±0.017a	0.564±0.005a	0.800±0.004a
	a5	0.755±0.045a	2.304±0.130a	0.891±0.050a	2.263±0.127a	0.530±0.048a	0.755±0.045a
	a6	0.808±0.012a	2.465±0.056a	0.954±0.022a	2.447±0.058a	0.578±0.013a	0.808±0.012a

CK, control line FN95–1702; a1, a2, a3, a4, a5, a6: cry1Ac sugarcane lines; values in the column followed by the same letters mean no statistically significant (P < 0.05 level, N = 3).

Using the functional diversity indices, there was no significant difference found at the elongation and maturating stages regardless of *cry1Ac* sugarcane or non-GM line type (Table 3). During the tillering stage, lines a3, a4 and a5 showed significant differences to the control line FN95–1702; however, the other three *cry1Ac* lines showed no significant difference to the control FN95–1702 (Table 3). The results indicate that the *cry1Ac* sugarcane had little effect on the functional diversity index of microorganisms in rhizosphere soil.

### Effect of *cry1Ac* transgenic sugarcane on enzyme activity in rhizosphere soil

**Figure 3:**
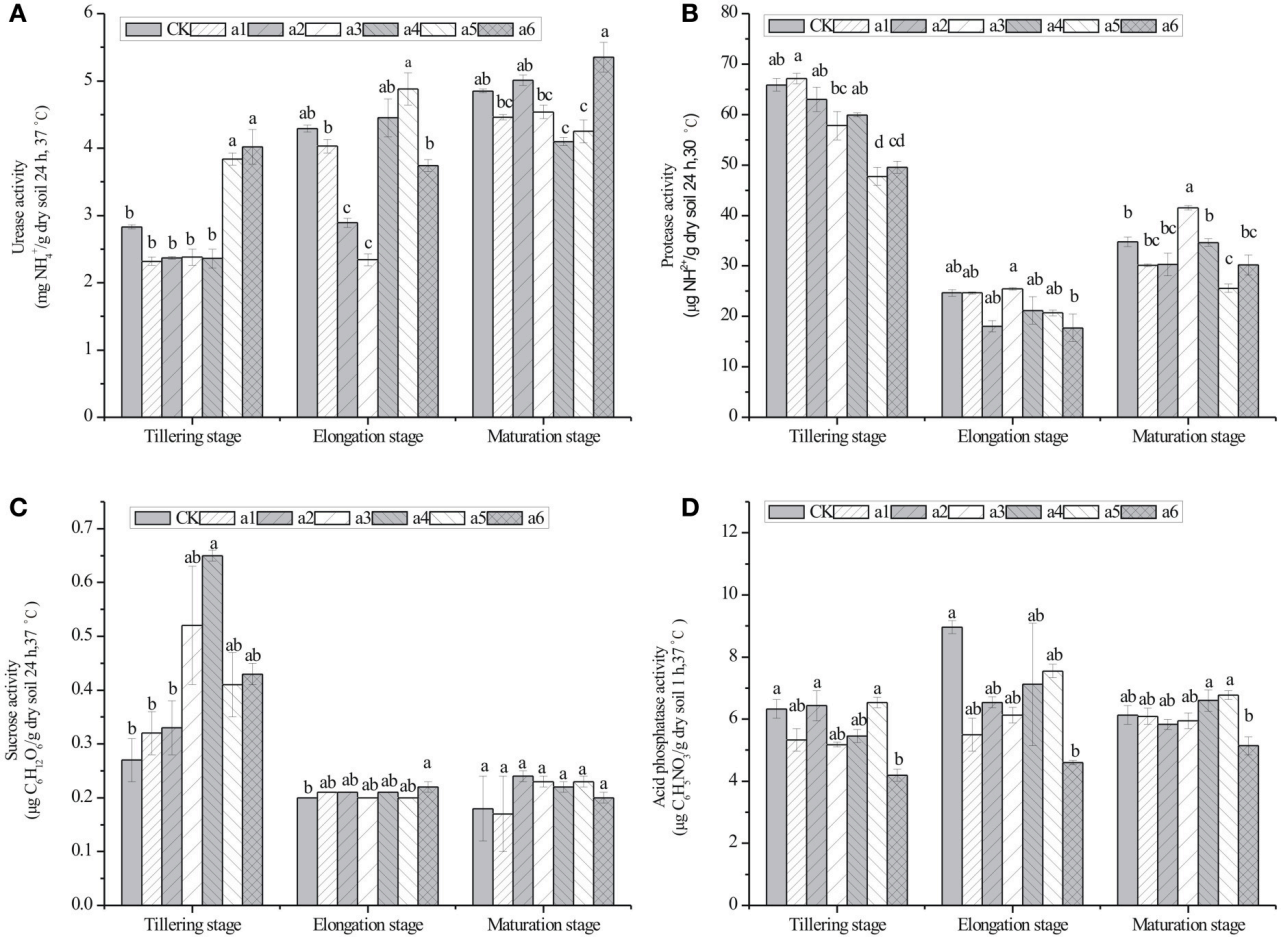
**Effects of *cry1Ac* sugarcane on activities of urease, protease, sucrose, and acid phosphatase in rhizosphere soil at the tillering, elongation and maturing stages. (A)** urease activity; **(B)** protease activity; **(C)** sucrase activity; **(D)** acid phosphatase activity. CK, control line FN95–1702. Vertical bars indicate standard error of the means (Mean ± SE, *N* = 3). The same letters represent no statistically significant differencewith the control line (*P* < 0.05 level). CK, control line FN95–1702. a1, a2, a3, a4, a5, a6: *cry1Ac* sugarcane lines.

The effects of *cry1Ac* transgenic sugarcane lines on the activity of four enzymes in rhizosphere soil are shown in Figure 3. The values of the urease activity in *cry1Ac* lines were from 2.32 ± 0.06 to 5.35 ± 0.22 mg ${\text{NH}}_{4}^{+}$ · g^−1^ dry soil (24 h, 37°C), while the control line had values from 2.83 ± 0.03 to 4.85 ± 0.03 mg ${\text{NH}}_{4}^{+}$ · g^−1^ dry soil (24 h, 37°C; Figure 3). The protease activity in *cry1Ac* lines shows the values from 17.71 ± 2.71 to 67.11 ± 1.04 μg ${\text{NH}}_{2}^{+}$ · g^−1^ dry soil (24 h, 30°C), while the control line varied from 24.64 ± 0.67 to 65.88 ± 1.23 μg ${\text{NH}}_{2}^{+}$ · g^−1^ dry soil (24 h, 30°C). The sucrase activity in *cry1Ac* lines varied from 0.20 ± 0.01 to 0.65 ± 0.01 μg C_6_H_12_O_6_· g^−1^ dry soil (24 h, 37°C), while the control line had values from 0.18 ± 0.06 to 0.27 ± 0.04 μg C_6_H_12_O_6_· g^−1^ dry soil (24 h, 37°C). The acid phosphatase activity in *cry1Ac* lines shows the values from 4.19 ± 0.01 to 7.55 ± 0.22 μg C_6_H_5_NO_3_· g^−1^ dry soil (1 h, 37°C), while the control line had values from 6.13 ± 0.30 to 8.96 ± 0.21 μg C_6_H_5_NO_3_· g^−1^ dry soil (1 h, 37°C).

Generally, as shown in Figure 3, most of the *cry1Ac* lines had no significant difference compared with the control line at the same stage. However, we also observed some instances of significant differences, such as the urease activity of a5 and a6 at the tillering stage.

Specifically, significant differences in the activity of urease were observed in the rhizosphere soil of a5 and a6 at the tillering stage, a2 and a3 at the elongation stage, a4 and a5 at the maturing stage. For the activity of protease, significant differences were observed in the rhizosphere of a5 at the tillering stage and a3 and a5 at the maturing stage. For the activity of sucrase, significant differences were observed in the rhizosphere of a4 at the tillering stage and a6 at the maturing stage. Finally, significant differences were observed in the activity of acid phosphatase in the rhizosphere of a6 at the tillering and elongation stages.

The variation pattern in the rhizosphere soil enzyme activities between GM sugarcane lines and non-GM sugarcane throughout their development showed no consistent trend (i.e. the soil enzyme activities of the same GM line changed with the development stage, or the soil enzyme activities at the same stage changed with different GM sugarcane lines). This suggests that the change in the soil enzyme activities may result from the differences in soil chemical properties such as pH, fertilizer addition or/and some other natural factors.

### DGGE and sequence analysis

**Figure 4:**
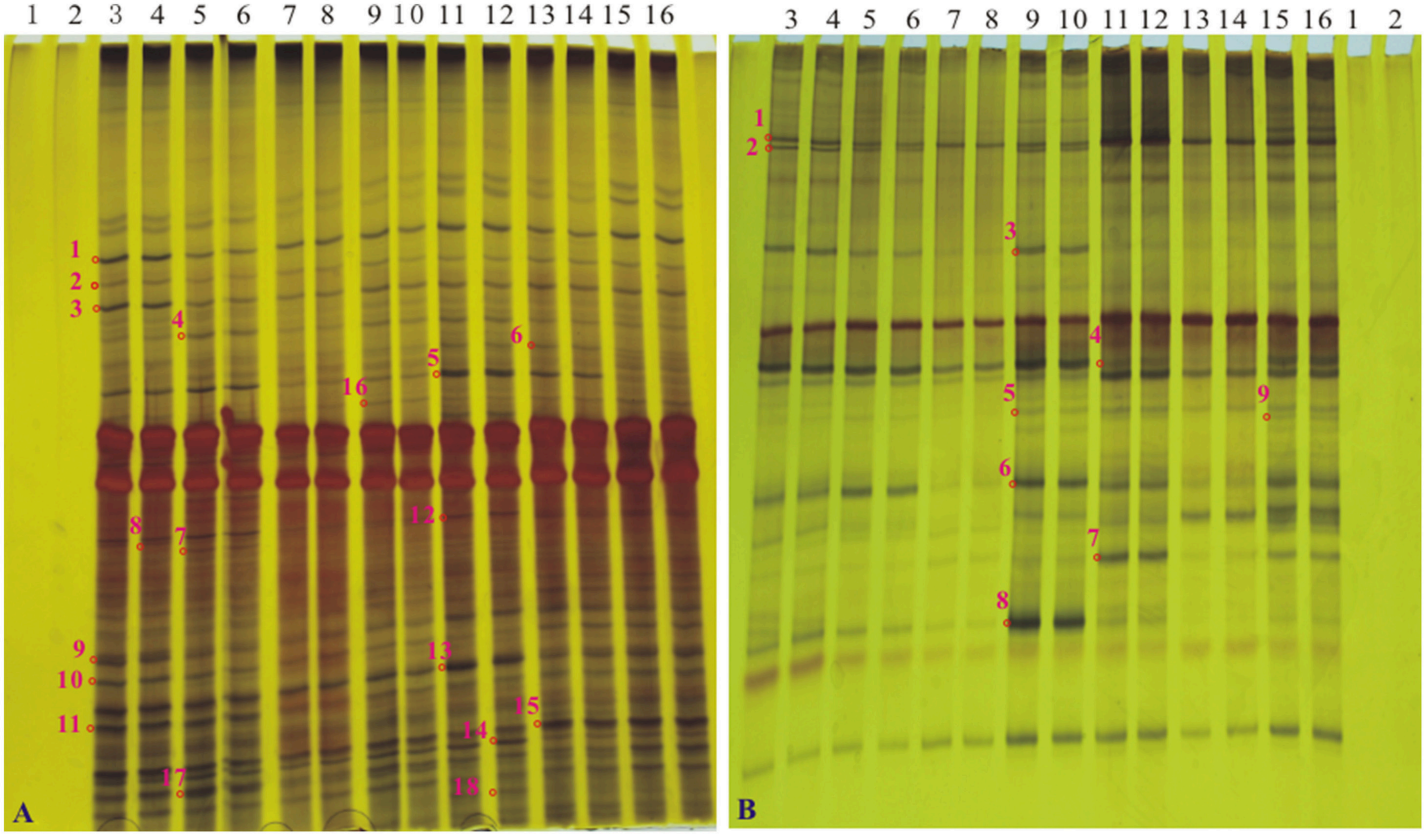
**The profiles of DGGE of soil bacterial *16S rDNA* gene and soil fungal *18S rDNA* gene at the tillering stage. (A)** the profile of DGGE of soil bacterial *16S rDNA*; **(B)** the profile of DGGE of soil bacterial *18S rDNA*. Lanes 1 and 2, H_2_O; lanes 3 and 4, sugarcane line FN95–1702; lanes 5 and 6, a1; lanes 7 and 8, a2; lanes 9 and 10, a3; lanes 11 and 12, a4; lanes 13 and 14, a5; lanes 15 and 16, a6. Bands 1–18 in A and bands 1–9 in B (marked as “o” with numbers along side it), The interested bands (present/absent or different intensity) were excised from the DGGE gel.

Silver-stained DGGE gel profiles represent the predominant bacterial or fungal community of the tillering soil samples cultivated the GM samples and the non-GM line FN95–1702 (Figure 4). The brightness of the bands correlates with the number of the bacterium or fungus. The DGGE profiles of *16S rDNA* and *18S rDNA* segments displayed the typical characteristics of soil samples. There were more than 20 bands for each sample of both *16S rDNA* and *18S rDNA*. Many equally intense bands, indicating the presence of a large number of equally abundant ribotypes, were observed for all soil samples. However, some strong or rather characteristic (present/absent or different intensity) bands (marked with red numbers in Figure 4) were observed in some samples. In total, 18 representative bands of bacterial *16S rDNA* PCR-DGGE and 9 representative bands of fungal *18S rDNA* PCR-DGGE were excised, cloned, and sequenced.

To determine whether communities of bacterium and fungus from three growth stages were significantly different in the six *cry1Ac* sugarcane and non-*GM* FN95–1702 samples, several diversity indices of the DGGE profiles were calculated on the basis of gray scanning (Tables 4, 5). The differences in the *16S rDNA* DGGE diversity index, including the Simpson's index, Shannon index, Brillouin index and McIntosh index, between the *cry1Ac* sugarcane samples and FN95–1702 control were not substantial at the tillering and elongation stages, while two *cry1Ac* sugarcane lines (a5 and a6) showed significant differences with the control line FN95–1702 at the mature stage (Table 4). No significant difference between *cry1Ac* sugarcane and non-GM samples was also found at the *18S rDNA* DGGE diversity index at the tillering and elongation stages, although line a4 was significantly different to the control line FN95–1702 (Table 5).

**Table 4:** **The *16S rDNA* DGGE diversity index analysis**.

**Periods**	**Lines**	**The ***16S rDNA*** DGGE diversity index (Mean ± SD)**				
		**Simpson's index**	**Shannon index**	**Evenness**	**Brillouin index**	**McIntosh index**
Tillering stage	CK	0.96±0.005a	4.92±0.054a	0.87±0.007a	4.19±0.039a	0.85±0.011a
	a1	0.96±0.003a	4.91±0.036a	0.88±0.004a	4.16±0.027a	0.86±0.006a
	a2	0.95±0.001a	4.8±0.0140a	0.87±0.000a	4.13±0.013a	0.84±0.003a
	a3	0.95±0.001a	4.84±0.023a	0.88±0.001a	4.12±0.032a	0.85±0.002a
	a4	0.95±0.001a	4.85±0.011a	0.87±0.002a	4.16±0.030a	0.85±0.002a
	a5	0.95±0.000a	4.81±0.025a	0.86±0.002a	4.16±0.020a	0.83±0.001a
	a6	0.95±0.003a	4.88±0.065a	0.87±0.007a	4.13±0.045a	0.84±0.007a
Elongation stage	CK	0.93±0.005a	4.44±0.119a	0.78±0.011a	3.80±0.111a	0.79±0.010a
	a1	0.94±0.030a	4.62±0.399a	0.85±0.068a	3.99±0.350a	0.83±0.064a
	a2	0.95±0.001a	4.70±0.032a	0.88±0.006a	4.08±0.025a	0.84±0.002a
	a3	0.93±0.008a	4.35±0.122a	0.83±0.023a	3.79±0.121a	0.80±0.015a
	a4	0.92±0.003a	4.15±0.056a	0.79±0.008a	3.61±0.058a	0.77±0.006a
	a5	0.95±0.002a	4.69±0.029a	0.86±0.005a	4.07±0.012a	0.84±0.005a
	a6	0.95±0.007a	4.75±0.043a	0.86±0.013a	4.07±0.016a	0.84±0.017a
Maturing stage	CK	0.92±0.008b	4.37±0.129ab	0.82±0.024b	3.77±0.104ab	0.78±0.015b
	a1	0.91±0.002b	4.32±0.027ab	0.82±0.005b	3.73±0.032ab	0.77±0.003b
	a2	0.90±0.006b	4.07±0.072bc	0.79±0.014b	3.53±0.067bc	0.75±0.010bc
	a3	0.90±0.003b	4.14±0.045bc	0.79±0.009b	3.59±0.040bc	0.74±0.006bc
	a4	0.91±0.001b	4.26±0.018ab	0.81±0.001b	3.68±0.016abc	0.77±0.002b
	a5	0.96±0.011a	4.63±0.137a	0.92±0.027a	4.05±0.124a	0.86±0.026a
	a6	0.86±0.005c	3.76±0.062c	0.75±0.013b	3.28±0.075c	0.68±0.007c

CK: control line FN95–1702; a1, a2, a3, a4, a5, a6: cry1Ac sugarcane lines; values in the column followed by the same letters mean no statistically significant (P < 0.05 level, N = 3).

**Table 5:** **The *18S rDNA* DGGE diversity index analysis**.

**Periods**	**Lines**	**The ***18S rDNA*** DGGE diversity index (Mean ± SD)**				
		**Simpson's index**	**Shannon index**	**Evenness**	**Brillouin index**	**McIntosh index**
Tillering stage	CK	0.96±0.006a	4.48±0.010a	0.94±0.022a	0.94±0.022a	0.87±0.015a
	a1	0.96±0.001a	4.53±0.028a	0.95±0.006a	0.95±0.006a	0.87±0.002a
	a2	0.95±0.009a	4.28±0.125a	0.92±0.021a	0.92±0.021a	0.84±0.021a
	a3	0.94±0.002a	4.19±0.011a	0.89±0.003a	0.89±0.003a	0.82±0.003a
	a4	0.95±0.008a	4.37±0.136a	0.93±0.018a	0.93±0.018a	0.85±0.019a
	a5	0.94±0.007a	4.09±0.069a	0.92±0.015a	0.92±0.015a	0.83±0.016a
	a6	0.95±0.011a	4.21±0.137a	0.91±0.024a	0.91±0.024a	0.84±0.024a
Elongation stage	CK	0.97±0.006a	4.73±0.175a	0.93±0.003a	4.11±0.161a	0.88±0.014a
	a1	0.96±0.004a	4.62±0.069a	0.92±0.006a	4.05±0.075a	0.87±0.010a
	a2	0.96±0.002a	4.46±0.053a	0.92±0.016a	3.92±0.056a	0.85±0.003a
	a3	0.96±0.000a	4.63±0.002a	0.93±0.004a	4.05±0.024a	0.87±0.001a
	a4	0.96±0.004a	4.56±0.124a	0.94±0.011a	3.99±0.116a	0.88±0.011a
	a5	0.96±0.002a	4.42±0.063a	0.91±0.008a	3.89±0.070a	0.86±0.005a
	a6	0.96±0.000a	4.45±0.020a	0.93±0.006a	3.85±0.055a	0.87±0.001a
Maturing stage	CK	0.96±0.001a	4.49±0.012ab	0.94±0.008ab	3.96±0.023a	0.87±0.002a
	a1	0.97±0.000a	4.70±0.008ab	0.94±0.002ab	4.09±0.012a	0.88±0.000a
	a2	0.96±0.003a	4.45±0.083ab	0.92±0.002b	3.91±0.068a	0.86±0.007a
	a3	0.97±0.000a	4.77±0.002a	0.95±0.000a	4.15±0.017a	0.89±0.001a
	a4	0.73±0.028b	2.02±0.150c	0.82±0.017c	1.89±0.136b	0.53±0.029b
	a5	0.95±0.003a	4.31±0.059b	0.91±0.007b	3.83±0.044a	0.84±0.008a
	a6	0.96±0.001a	4.47±0.031ab	0.92±0.008b	3.92±0.014a	0.86±0.003a

CK, control line FN95–1702; a1, a2, a3, a4, a5, a6: cry1Ac sugarcane lines; values in the column followed by the same letters mean no statistically significant (P < 0.05 level, N = 3).

**Figure 5:**
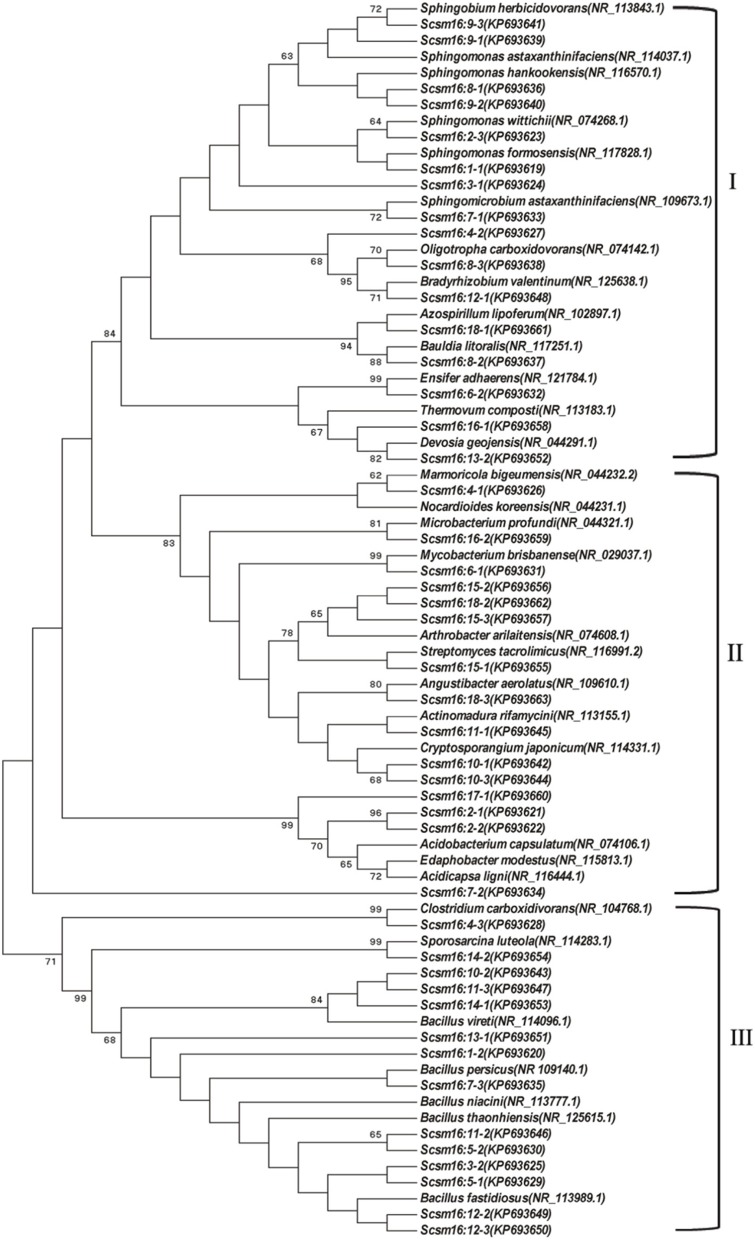
**Phylogenetic tree showing the relationship between the individual bacterial *16S rDNA* genes from the cultivated soil under *cry1Ac* and non-GM sugarcane**. The numbers before transverse line represent different band marked in “o” with numbers (in Figure 4A), while the numbers after the transverse line represent different positive sub-clones. I, Proteobacteria; II, Actinobacteria; III, Firmicutes.

**Figure 6:**
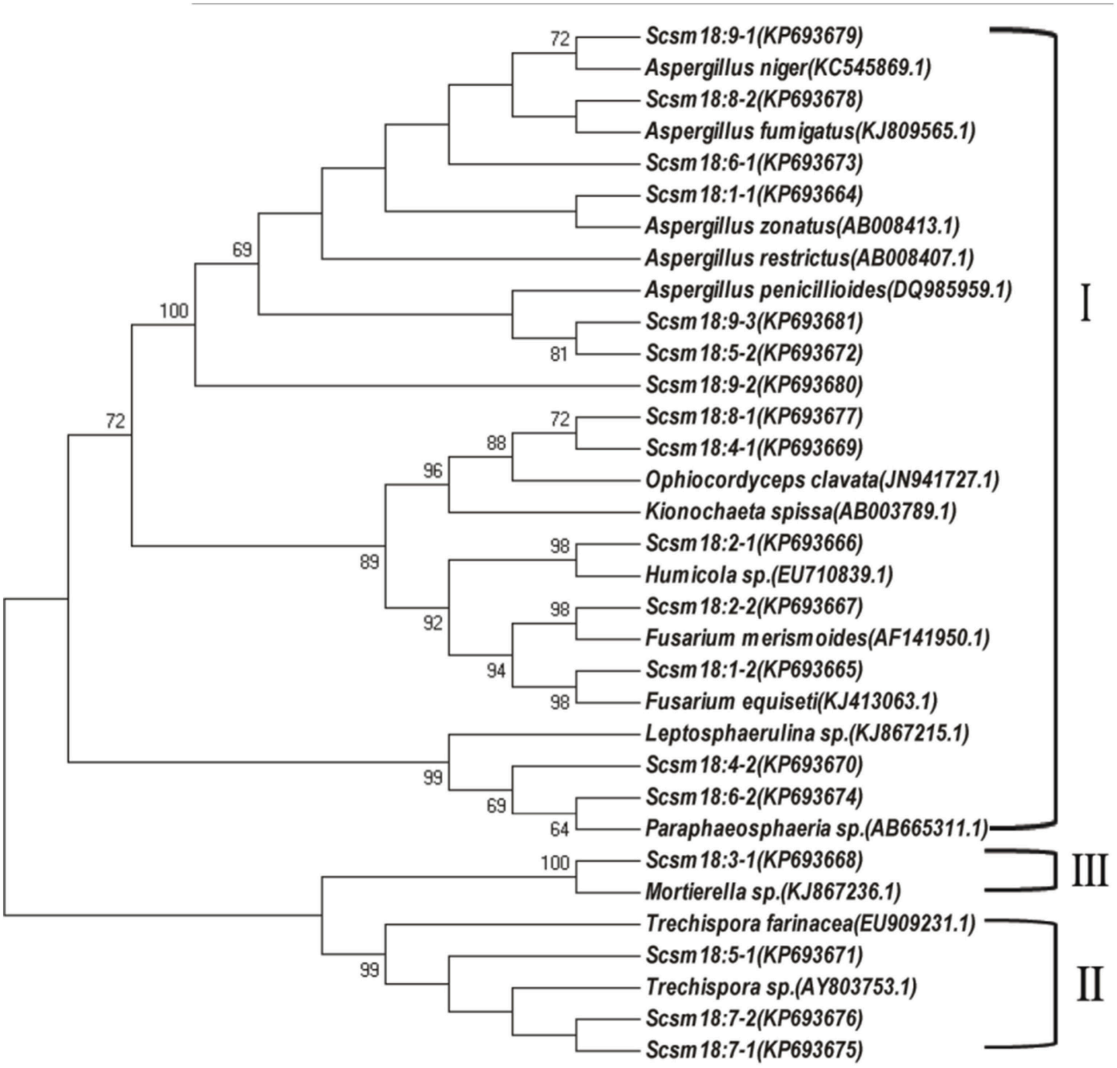
**Phylogenetic tree showing the relationship between individual fungal *18S rDNA* genes in the cultivated soil *cry1Ac* and non-GM sugarcane**. The figure before the transverse line represents different band marked in “o” with numbers (in Figure 4B), while the figure behind the transverse line represent different positive sub-clones. I, Ascomycetes; II, Basidiomycetes; III, unknown phylum cluster.

DGGE followed by cloning techniques is a practicable method to understand the complex community of soil microbes. Based on NCBI blast, the most similar strains or the closest neighbors of the nucleotide sequences of bacteria and fungi in the soil in which GM and non-GM sugarcane cultivated are shown in Supplementary Tables 1, 2. Most of the DGGE bands yielded more than one sequence, which presented one specific strain. The results revealed that the species diversity of bacteria in the rhizosphere soil was higher than that of fungi. Because the reconstruction of the phylogenetic tree of organisms is one of the most important issues in the study of evolution, we constructed the phylogenetic tree to indicate the relationship among individual bacterial *16S rDNA* genes (Figure 5) or fungal *18S rDNA* genes (Figure 6) from the soil under *cry1Ac* sugarcane and non-GM sugarcane cultivation. For bacteria there are 10 and 9 strains clustered closely to *Bacillus* and *Sphingomonas*, respectively, while for fungus there are more than 12 and 7 strains clustered closely to *Aspergillus* and *Trechispora*, respectively (Supplementary Tables 1, 2). The result of the phylogenetic tree analysis based on subclone sequences and the most similar species through the blast showed that all these bacteria were members of three distinct phyla (Figure 5). The observed fungi were members of three distinct phyla (Figure 6). The DGGE and the sequence data of *16S rDNA* indicated that most of bacteria predominantly belonged to the phyla Proteobacteria, Actinobacteria and Firmicutes, whilst isolated fungal sequences belonged primarily to the phyla Ascomycetes and Basidiomycetes. (Supplementary Tables 1, 2 and Figures 5, 6).

## Discussion

Crop residues are the major sources of C and N in cultivated soil, and root exudates may affect organism composition in the rhizosphere (Icoz and Stotzky, 2008). Because of the production of foreign proteins in all parts of the plant, GM crops have the potential to change the microbial dynamics, biodiversity, and essential ecosystem functions in soil (Icoz and Stotzky, 2008). Risk assessment of GM crops impact on soil organisms is considered to be crucial (Icoz and Stotzky, 2008).

The possibility of exogenic gene flow from GM-crops to related wild species or to associated weeds is one of the major concerns related to the ecological risks of the commercial release of transgenic plants (Messeguer, 2003). Therefore, gene flow or the possibility of gene flow must be considered when assessing the potential environmental impact of cultivating GM plants (Chandler and Dunwell, 2008). Although gene flow is usually mediated by pollen and seeds (Oddou-Muratorio et al., 2001; Ward et al., 2005; Scorza et al., 2013), some gene flow is a result of asexual propagation, which would typically result in the long-term survival and the spread of plant residues to the soil or to other new plants (Chandler and Dunwell, 2008). For sugarcane, which is an asexual propagation crop, gene flow is certainly related partly to asexual propagation. Gene flow has low frequency or probability even under natural conditions, just as beneficial mutations under the appropriate natural conditions (Slatkin, 1985). Sugarcane with very strict blossoming requirements is an industrial material crop and is propagated by vegetative stalks and by micropropagation *in vitro* in its commercial production. In China, there is almost no flowering or due to pollen sterility, and sugarcane seed derived from blossom hybridization is carried out only in Yacheng, Hainan province or in Ruili, Yunnan province (Chen et al., 2011). Thus, gene flow would most probably be observed by asexual propagation or introgression between sugarcane lines and its wild relatives or soil microorganisms. In our study of the soil risk assessment, PCR results showed no detectable gene flow, which suggests that the *cry1Ac* sugarcane has no gene flow in rhizosphere soil.

Any impact that *cry1Ac* sugarcane has on the rhizosphere microbial community could have either positive or negative effects on plant growth, and in turn ecosystem sustainability (Dunfield and Germida, 2004). The effect of GM-plant roots on the rhizosphere soil microorganisms is usually investigated using the classic plate counting method (Brusetti et al., 2005; Houlden et al., 2008; D'angelo-Picard et al., 2011; Li et al., 2011), community-level physiological profiles (CLPP) (Griffiths et al., 2005), Biolog EcoPlate™ (Dunfield and Germida, 2004; Chaudhry et al., 2012; Janniche et al., 2012; Lv et al., 2014), single-strand conformation polymorphism (SSCP), terminal-restriction fragment length polymorphism (T-RFLP), DGGE (Griffiths et al., 2005; D'angelo-Picard et al., 2011), or PCR-DGGE (Crecchio et al., 2007; Lv et al., 2014), and even metagenomics and high-throughput sequencing (HTS) (Cleary et al., 2012). Though having its own limitations, compared with culture dependent methods, modern molecular techniques have been used widely since they enable scientists to obtain more realistic information about microbes in the environment (Cleary et al., 2012). For example, though the CLPP method often requires certain types of multivariate analyses for interpretation, which may be a challenge and requires a significant statistical background along with an understanding of the inferences and biases each multivariate analysis method incurs, the CLPP method is a straight forward laboratory protocol and a popular method to characterize and track changes in heterotrophic bacterial communities (Weber and Legge, 2009).

DGGE, which was introduced into microbiology by Muyzer and Smalla (1998), has been widely applied for profiling the structure of bacterial communities and for the analysis of the composition of a range of microbial groups (Mocali et al., 2005; Crecchio et al., 2007; Cleary et al., 2012; Wu et al., 2014). Mocali et al. (2005) assessed the effects of *Bt* corn and non-*Bt* corn lines on soil ecosystems by means of DGGE analysis of *16S rDNA* genes. Wu et al. (2014) analyzed the impact of transgenic wheat N12-1 on bacterial and fungal community diversity in rhizosphere soil using PCR-DGGE. Compared to HTS, DGGE has the limitation of low coverage. However, PCR-DGGE is an appropriate option especially when the research objective is to compare the organism diversity during a time process or over different sites (Mocali et al., 2005; Vaz-Moreira et al., 2013). In addition, in combination with sub-clone and sequencing, DGGE can be a useful protocol to assess phylogenetic diversity. Therefore, in the current study, we selected the classic plate counting method, Biolog EcoPlate™ and PCR-DGGE as our protocol to assess the effect of *cry1Ac* sugarcane on the rhizosphere soil microorganisms. In the present study, the microbial communities of the *cry1Ac* sugarcane rhizosphere soils were compared with that of non-transgenic sugarcane to assess the ecological effect on structural diversity of planting the *cry1Ac* sugarcane. We found that the population of culturable microorganisms was not significantly different to the control line FN95–1702 at each growth stage. Meanwhile, the results of the Biolog EcoPlate™ revealed that spatial and temporal changes in community-level show a consistent trend between the *cry1Ac* sugarcane and the non-transgenic control line based the AWCD values, PCA and the functional diversity index. The DGGE analysis showed consistent results with the diversity index fromthe Biolog EcoPlate™. The band patterns of the DGGE profiles displayed the typical characteristics of soil samples. From the sequence data and phylogenetic tree analysis of DGGE, we found that the *cry1Ac* sugarcane soil fungi predominantly belonged to the Ascomycetes and Basidiomycetes, while isolated bacterial sequences belonged primarily to the Proteobacteria, Actinobacteria, and Firmicutes. This is in accordance with previous studies showing Basidiomycetes and Ascomycete to be predominant fungi in agricultural and grassland soils (Xu et al., 2012), which seems to suggest that comparison with non-GM sugarcane, *cry1Ac* sugarcane roots have no more major on the rhizosphere microbial community, especially the main predominant culturable microbial groups. In combination with the diversity index analysis, sequence data and phylogenetic tree analysis, we know that the richness of the bacteria and fungi is roughly constant, while there is a shift in the dominant species which we suggest is largely due to sugarcane growth or other climatic factors, since the UPGMA (unweighted pair group averages) analysis, based on dice coefficients, revealed that the patterns of each sample clustered separately, while the two replications clustered together (Supplementary Figure 2).

Soil enzymes are usually studied in risk assessment of transgenic plants because of their involvement in soil nutrient cycling (Nakatani et al., 2014). Analysis of soil enzyme activities is not only used as early and sensitive indicators of management-induced changes in soil fertility and stress, but also as indicators of productivity, sustainability, and pollution of the crops (Nannipieri et al., 1994). Soil enzymes play an important role in the transformation of nutrients, organic matter decomposition, degradation, and remediation of pollutants (Li X. et al., 2014). Li X. et al. (2014) shows that soil urease activities are significantly correlated to the nutrition content, which can be used as the biological index to evaluate the soil fertility. Soil protease, sucrose, and acid phosphatase are important in the N-cycling, C-cycling and P-cycling, respectively, and also in soil texture and other soil characteristics (Li P. et al., 2014). Soil enzymes catalyze decomposition in the soil of matter, from microorganisms, plants, animals, and living secretion of debris (Li X. et al., 2014). In the current study, the data obtained from the selected soil enzymes indicated that the GM sugarcane lines had few negative effects on the soil urease, protease, sucrase and acid phosphatase, when compared to the non-GM sugarcane line, although some transient or even significant differences were observed. This result is consistent with the previous study (Fang et al., 2012). Shen et al. (2006) reported that there were few instances of significance in urease and protease activities between *Bt* and non-*Bt* cottons at any of the growth stages. Fang et al. (2012) showed that there are some significant differences in soil enzyme (catalase, urease, neutral, phosphatase and invertase) activities between transgenic *Bt* rice lines.

Laboratory and field studies suggest that differences in the persistence of the foreign proteins appear to be the result primarily of differences in microbial activity (Heuer et al., 2002; Houlden et al., 2008; Icoz and Stotzky, 2008) The variation pattern of the rhizosphere soil enzyme activities between GM plant lines and non-GM plants throughout their development may be affected by the differences in soil chemical properties such as pH, clay mineral composition, physicochemical characteristics or/and some other natural factors (Icoz and Stotzky, 2008; Chen et al., 2012). Other natural factors are expected to cause variation in the effect of *cry1Ac* sugarcane and non-transgenic lines on rhizosphere soil enzyme activity. These factors include seasonal changes, rainfall amounts and distribution as suggested in previous reports (Icoz and Stotzky, 2008; Lv et al., 2014). Some of these natural factors, play an important role in governing the population of rhizosphere microbial communities, and would mask the effect of plant species on bacterial community activity and also on resource utilization potential. Such factors are summarized as follows: the plant developmental stage, soil type, season (e.g., temperature, water tension), crop species (e.g., chemical composition, C: N ratio, plant part), and crop management practices (Heuer et al., 2002; Houlden et al., 2008).

The present study revealed that the *cry1Ac* sugarcane lines may not affect the microbial community structure and functional diversity of the rhizosphere soil and have few negative effects on soil enzymes, based on one crop season. Such studies are important to determine the potential risks of *cry1Ac* sugarcane. This is the first comprehensive study on risk assessment of *cry1Ac* sugarcane on rhizosphere soil ecosystems.

## Author contributions

Conceived and designed the experiments: DZ, LX, and YQ. Performed the experiments: DZ, SG, JG, JL, and QY. Analyzed the data: DZ, LX, and YQ. Wrote the paper: DZ, LX, and YQ. Revised and approved the final version of the paper: LX, YQ.

### Conflict of interest statement

The authors declare that the research was conducted in the absence of any commercial or financial relationships that could be construed as a potential conflict of interest.
