# Supplementary material for: Cry1Ac Transgenic Sugarcane Does Not Affect the Diversity of Microbial Communities and Has No Significant Effect on Enzyme Activities in Rhizosphere Soil within One Crop Season
Source: Front Plant Sci. 2016 Mar 8;7:265. doi: 10.3389/fpls.2016.00265 (PMC4781841; doi:10.3389/fpls.2016.00265)
Supplement: Supplementary file 1 [file Table1.PDF]

Supplementary Material

Cry1Ac Transgenic Sugarcane Does Not Affect the Diversity of Microbial Communities and Has No Significant Effect on Enzyme Activities in Rhizosphere Soil within One Crop Season

Dinggang Zhou, Liping Xu*, Shiwu Gao, Jinlong Guo, Jun Luo, Qian You, Youxiong Que*

***Correspondence**: Liping Xu and Youxiong Que, Key Laboratory of Sugarcane Biology and Genetic Breeding, Fujian Agriculture and Forestry University, Ministry of Agriculture, Fuzhou 350002, China . E-mail: xlpmail@126.com, queyouxiong@126.com

1. Supplementary Figures and Tables

1.1. Supplementary Figures

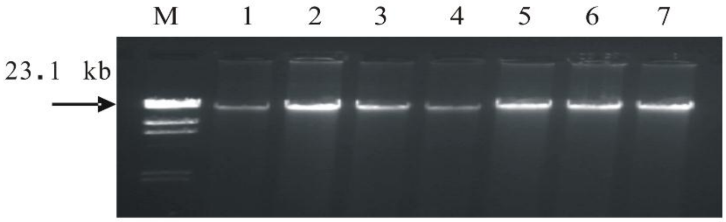

Supplementary Figure 1. The gel electrophoresis of the total genomic DNA from microorganisms in soil. M：λ *Hin*d Ⅲ DNA ladder; 1: FN95-1702 (control line); 2~7: a1, a2, a3, a4, a5 and a6 (*cry1Ac* transgenic lines) in turn.

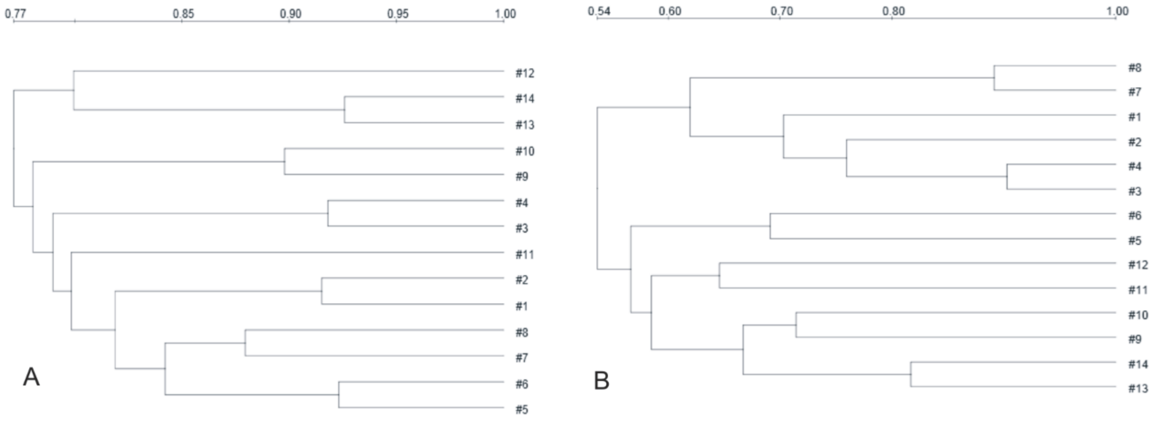

Supplementary Figure 2. UPGMA analysis of the DGGE gel profiles of *16S rDNA* and *18S rDNA* from the soil of GM and non-GM sugarcane cultivated basing on dice coefficient. (A) UPGMA analysis of *16S rDNA* in soil bacteria; **(B)** UPGMA analysis of *18S rDNA* in soil fungi. “#” in this figure means sample. Samples 1 and 2: sugarcane line FN95-1702; samples 3 and 4: a1; samples 5 and 6: a2; samples 7 and 8: a3; samples 9 and 10: a4; samples 11 and 12: a5; samples 13 and 14: a6, two repeats, respectively.

1.2. Supplementary Tables

Supplementary Table 1. The most similar bacterial strains in GM and non-GM sugarcane cultivated rhizosphere soil based on NCBI blast

Band number	Division	Closest cultivable species	Accession No.	Sequence similarity (%)
Scsm16:1-1	Proteobacteria	*Sphingomonas formosensis*	NR_117828.1	100
Scsm16:1-2	Firmicutes	*Bacillus thaonhiensis*	NR_125615.1	99
Scsm16:2-1	Acidobacteria	*Acidicapsa ligni*	NR_116444.1	98
Scsm16:2-2	Acidobacteria	*Edaphobacter modestus*	NR_115813.1	98
Scsm16:2-3	Proteobacteria	*Sphingomonas wittichii* RW1	NR_074268.1	99
Scsm16:3-1	Proteobacteria	*Sphingomonas formosensis*	NR_117828.1	98
Scsm16:3-2	Firmicutes	*Bacillus thaonhiensis*	NR_125615.1	98
Scsm16: 4-1	Actinobacteria	*Marmoricola bigeumensis*	NR_044232.2	99
Scsm16:4-2	Proteobacteria	*Oligotropha carboxidovorans* OM5	NR_074142.1	98
Scsm16:4-3	Firmicutes	*Clostridium carboxidivorans* P7	NR_104768.1	99
Scsm16:5-1	Firmicutes	*Bacillus fastidiosus*	NR_113989.1	99
Scsm16:6-1	Firmicutes	*Mycobacterium brisbanense*	NR_029037.1	99
Scsm16:6-2	Actinobacteria	*Ensifer adhaerens*	NR_121784.1	100
Scsm16:7-1	Proteobacteria	*Sphingomicrobium astaxanthinifaciens*	NR_109673.1	98
Scsm16:7-2	Proteobacteria	*Nocardioides koreensis*	NR_044231.1	98
Scsm16:7-3	Actinobacteria	*Bacillus persicus*	NR_109140.1	100
Scsm16:8-1	Firmicutes	*Sphingomonas hankookensis*	NR_116570.1	100
Scsm16: 8-2	Proteobacteria	*Bauldia litoralis*	NR_117251.1	100
Scsm16:8-3	Proteobacteria	*Oligotropha carboxidovorans* OM5	NR_074142.1	100
Scsm16:9-1	Proteobacteria	*Sphingomonas astaxanthinifaciens*	NR_114037.1	99
Scsm16:9-2	Proteobacteria	*Sphingomonas hankookensis*	NR_116570.1	100
Scsm16:9-3	Proteobacteria	*Sphingobium herbicidovorans*	NR_113843.1	99
Scsm16:10-1	Proteobacteria	*Cryptosporangium japonicum*	NR_114331.1	98
Scsm16:10-2	Actinobacteria	*Bacillus vireti* LMG 21834	NR_114096.1	99
Scsm16:10-3	Firmicutes	*Cryptosporangium japonicum*	NR_114331.1	99
Scsm16:11-1	Actinobacteria	*Actinomadura rifamycini*	NR_113155.1	99
Scsm16:11-2	Actinobacteria	*Bacillus niacini*	NR_113777.1	99
Scsm16:11-3	Firmicutes	*Bacillus vireti* LMG 21834	NR_114096.1	100
Scsm16:12-1	Firmicutes	*Bradyrhizobium valentinum*	NR_125638.1	100
Scsm16:12-2	Proteobacteria	*Bacillus fastidiosus*	NR_113989.1	99
Scsm16:13-1	Firmicutes	*Bacillus niacini*	NR_113777.1	99
Scsm16:13-2	Firmicutes	*Devosia geojensis*	NR_044291.1	98
Scsm16:14-1	Firmicutes	*Bacillus soli*	NR_114095.1	100
Scsm16:14-2	Proteobacteria	*Sporosarcina luteola*	NR_114283.1	99
Scsm16:15-1	Firmicutes	*Streptomyces tacrolimicus*	NR_116991.2	99
Scsm16:15-2	Firmicutes	*Arthrobacter arilaitensis* Re117	NR_074608.1	100
Scsm16:16-1	Actinobacteria	*Thermovum composti*	NR_113183.1	98
Scsm16:16-2	Actinobacteria	*Microbacterium profundi*	NR_044321.1	98
Scsm16:17-1	Actinobacteria	*Acidobacterium capsulatum*	NR_074106.1	98
Scsm16:18-1	Proteobacteria	*Azospirillum lipoferum* 4B	NR_102897.1	98
Scsm16:18-2	Actinobacteria	*Arthrobacter arilaitensis* Re117	NR_074608.1	99
Scsm16:18-3	Acidobacteria	*Angustibacter aerolatus*	NR_109610.1	99

Note: the numbers before transverse line represent different band marked in “**o**” with numbers (in **Figure 4A**), while the numbers after transverse line represent different positive sub-clones.

Supplementary Table 2. The most similar fungal strains in GM and non-GM sugarcane lines cultivated rhizosphere soil based on NCBI blast

Band number	Division	Closest cultivated species	Accession No.	Sequence similarity (%)
Scsm18:1-1	Ascomycota	*Aspergillus zonatus*	AB008413.1	98
Scsm18:1-2	Ascomycota	*Fusarium equiseti*	KJ413063.1	100
Scsm18:2-1	Ascomycota	*Humicola sp.* 18002	EU710839.1	100
Scsm18:2-2	Ascomycota	*Fusarium merismoides*	AF141950.1	100
Scsm18:3-1	Fungal	*Mortierella sp.* BlackDot	KJ867236.1	99
Scsm18:4-1	Ascomycota	*Kionochaeta spissa*	AB003789.1	98
Scsm18:4-2	Ascomycota	*Leptosphaerulina sp.* DBCMVB	KJ867215.1	99
Scsm18:5-1	Basidiomycota	*Trechispora farinacea*	EU909231.1	100
Scsm18:5-2	Ascomycota	*Aspergillus penicillioides*	DQ985959.1	99
Scsm18:6-1	Ascomycota	*Aspergillus fumigatus*	KJ809565.1	99
Scsm18:6-2	Ascomycota	*Paraphaeosphaeria sp.* E5-3C	AB665311.1	100
Scsm18:7-1	Basidiomycota	*Trechispora sp.* PBM418	AY803753.1	100
Scsm18:7-2	Basidiomycota	*Trechispora farinacea*	EU909231.1	99
Scsm18:8-1	Ascomycota	*Ophiocordyceps clavata*	JN941727.1	99
Scsm18:8-2	Ascomycota	*Aspergillus fumigatus*	KJ809565.1	100
Scsm18:9-1	Ascomycota	*Aspergillus niger*	KC545869.1	99
Scsm18:9-2	Ascomycota	*Aspergillus restrictus*	AB008407.1	98
Scsm18:9-3	Ascomycota	*Aspergillus penicillioides*	DQ985959.1	100

Note: the numbers before transverse line represent different band marked in "**o**" with numbers (in **Figure 4B**), while the numbers after transverse line represent different positive sub-clones.
